# Biomarker metabolites capturing the metabolite variance present in a rice plant developmental period

**DOI:** 10.1186/1471-2229-5-8

**Published:** 2005-05-31

**Authors:** Lee Tarpley, Anthony L Duran, Tesfamichael H Kebrom, Lloyd W Sumner

**Affiliations:** 1 Texas A&M Agricultural Research and Extension Center, 1509 Aggie Dr, Beaumont, Texas, 77713, USA; 2 Soil and Crop Sciences Department, Texas A&M University, College Station, Texas, USA; 3 Analytical Research Laboratories, Oklahoma City, Oklahoma, USA; 4 Samuel Roberts Noble Foundation, Ardmore, Oklahoma, USA

## Abstract

**Background:**

This study analyzes metabolomic data from a rice tillering (branching) developmental profile to define a set of biomarker metabolites that reliably captures the metabolite variance of this plant developmental event, and which has potential as a basis for rapid comparative screening of metabolite profiles in relation to change in development, environment, or genotype. Changes in metabolism, and in metabolite profile, occur as a part of, and in response to, developmental events. These changes are influenced by the developmental program, as well as external factors impinging on it. Many samples are needed, however, to characterize quantitative aspects of developmental variation. A biomarker metabolite set could benefit screening of quantitative plant developmental variation by providing some of the advantages of both comprehensive metabolomic studies and focused studies of particular metabolites or pathways.

**Results:**

An appropriate set of biomarker metabolites to represent the plant developmental period including the initiation and early growth of rice tillering (branching) was obtained by: (1) determining principal components of the comprehensive metabolomic profile, then (2) identifying clusters of metabolites representing variation in loading on the first three principal components, and finally (3) selecting individual metabolites from these clusters that were known to be common among diverse organisms. The resultant set of 21 biomarker metabolites was reliable (*P *= 0.001) in capturing 83% of the metabolite variation in development. Furthermore, a subset of the biomarker metabolites was successful (*P *= 0.05) in correctly predicting metabolite change in response to environment as determined in another rice metabolomics study.

**Conclusion:**

The ability to define a set of biomarker metabolites that reliably captures the metabolite variance of a plant developmental event was established. The biomarker metabolites are all commonly present in diverse organisms, so studies of their quantitative relationships can provide comparative information concerning metabolite profiles in relation to change in plant development, environment, or genotype.

## Background

Variation in crop development due to genotype and environment strongly impacts yield. Increases in crop production efficiency are needed on a global basis because of projected expanding human populations coincident with regional decreases in area of arable land [1,2]. "An understanding of crop responses to environment will provide the fundamental basis for developing methods for achieving these increases in efficiency" (Hall,[2]). Plants interact with environment in both chemical and physical ways, but we have very little systematic understanding of how the plant responds chemically during development and in developmental response to environment [3,4]. This lack of knowledge of the broad changes in metabolite patterns during development limits our efficiency to manipulate the cellular or molecular aspects of plant development with intent to influence yield or sustainability of production.

Recent advances in plant metabolomics, that is large-scale phytochemical analysis of plants [5-7], are paving the way for identifying broad changes in metabolite patterns. Metabolomics has typically been used to characterize the comprehensive changes due to specific environmental or genetic perturbations [6]. Gas chromatography-mass spectrometry (GC-MS) methods currently are being used in many of the metabolomics studies and can provide an accurate and reproducible quantitative and qualitative assessment of a large complement of the metabolome [5,8,7]. A potential disadvantage of the GC-MS methods lies in the serial processing of samples. The time required to analyze large sample numbers can be lengthy for some studies.

Many samples are needed to characterize quantitative aspects of developmental variation. In these situations, methods that use parallel processing of samples to allow high-throughput assay would complement the traditional comprehensive, but serial, procedures, such as GC-MS. A potential disadvantage of the parallel processing methods lies in their dependence on predetermination of the metabolites to be assayed, which presents a possible bias in observed metabolite patterns.

The use of biomarker metabolites is common in many biological fields, including clinical chemistry. Foyer et al. [9] have recently proposed the use of certain amino acids or combinations of them as biomarker metabolites of several metabolic processes or states of plants. In the clinical-chemistry approach, the metabolites are typically chosen based on their diagnostic value, whereas in our study an approach was sought that combined the advantages of the diagnostic approach and the comprehensive metabolomic approach. The comprehensiveness is approached when the set of biomarkers captures much of the variance of the metabolome. The diagnostic value is approached through interpretation of the pattern of the biomarkers relative to each other, and the shifts or distortions in this pattern under various conditions. We anticipate that a biomarker metabolite set constructed through data reduction methods will substantially overlap or capture biomarker sets developed through knowledge of plant physiology.

Representatives from clusters of metabolites can probably capture much of the metabolite variance of a metabolomics study because multiple correlations among metabolites are commonly observed in metabolomics studies [10]. If a set of representatives could be identified for which: (1) the elements (metabolites) represented much of the metabolite variance within a study potentially impacting the improvement of crop production efficiency, (2) the elements were relatively independent of each other, and (3) the elements were common and found in any typical plant sample, then the resultant set of biomarker metabolites could be used in comparative screening of metabolite patterns of plant developmental periods, of plant response to specific environmental factors, or of genotypes in set conditions, and could provide a complementary tool of the comprehensive metabolomic technologies and of diagnostic biomarker approaches.

Metabolite composition is expected to vary consistently in response to development and environment. Core primary metabolites are known to provide good metabolite discrimination between genotypes [11]. This is expected because their quantities are typically affected by many genetic changes, i.e. they are involved in highly regulated activities. Also likely would be an effect on their quantities due to developmental change partially triggered by the internal programming influencing development, and by the need for certain metabolic transitions to occur with a change in growth pattern [9]. Many of these core primary or central metabolites show significant change in response to environmental conditions [12,13]. Knowledge of variation in central metabolism is furthermore considered fundamental for the progress of metabolic engineering [14], indicating a broad belief in its consistent and impacting variation. Primary and central metabolites need to change differentially in development and in response to environment because of biological reasons, and have been demonstrated to do so in a variety of organisms (the examples above include higher plants and *Escherichia coli*). In addition, a fair amount of general knowledge and assay procedures exist for these metabolites. The likelihood of meeting the three requirements itemized in the above paragraph was considered good, if the following simple procedure was used: 1) partition the variance in metabolites of the developmental event into independent components, 2) ensure that the variation within each of the major components was represented, and then 3) identify a minimal set of central (or nearly central) metabolites that satisfied these conditions.

The utility of a biomarker metabolite set for a developmental study depends on the ability of the biomarkers to provide a snapshot of an aspect of plant development. The ability of the biomarkers to faithfully represent the pattern of variation among the tissues sampled at various locations within the plant and at different plant ages provides a type of internal validation. An appropriate representation of the pattern of relationships among the tissue samples of this study is the set of correlations, based on the metabolite data, existing among them. The reliability of the biomarker metabolite set to capture the metabolite variance can thus be based on the ability to discern the same pattern of relationships among the tissues based on the correlations among them relative to the relationships based on correlations utilizing a more comprehensive metabolite dataset.

If a reliable, validated set of biomarker metabolites could be developed, then a final objective was to provide a demonstration of the type of output from comparative screening of metabolite patterns.

The metabolomics dataset used for development of the proposed biomarker metabolite set examined metabolite composition during initial tiller development of rice (*Oryza sativa *L.). Tillering is a major yield component of rice, as well as of wheat and many of the other small grains, because the number of tillers per land area strongly influences the number of panicles (heads of grain) per land area. Tiller initiation is sensitive to genotype and environmental effects. Environmental factors affecting tiller initiation include most known to affect plant developmental events, such as radiation quantity and spectral quality, adequacy of nutrition, extent of oxidative stress, and presence of growth inhibitors. A longer-term goal of our project is to understand the commonalities and differences in how these factors affect tiller initiation, so that schemes can be developed to minimize their effects and increase the consistency and manipulability of rice tillering and thus rice crop yield and quality.

## Results and discussion

First, some interpretation is provided of the principal components of the metabolite space in rice tiller development. This includes some examination of the patterns in metabolite loadings on Principal Component 1. Next, the results from a metabolite clustering based on the ranked principal component loadings are provided. This section includes the resulting selection of the biomarker metabolites, and a check of their relative independence. The next section discusses the physiological relevance of the biomarker metabolites with a focus on their loadings on the top three principal components. After this, some internal validation is provided by analyzing the reliability of the biomarker set to represent the pattern of metabolite variation observed among the tissues. An external validation is then provided by testing the ability of individual biomarkers to predict the changes in concentration of other metabolites in response to an environmental variable. Finally, a type of output from comparative screening of metabolite patterns is demonstrated.

### Interpretation of principal components of the metabolite space in rice tiller development

The metabolite profiles from the rice tiller development study were redistributed into independent subsets through the application of principal component analysis of the metabolite space (as opposed to plant developmental series space). Principal components in standardized centered metabolite space were determined, thus the results are based on analysis performed on the magnitude and pattern of the variation in concentrations of the individual metabolites (rather than on their absolute concentrations). The first five principal components, which explained 83% of the total metabolite variance (the first three principal components explained 62% of the total), were evaluated in this study.

**Figure 1:**
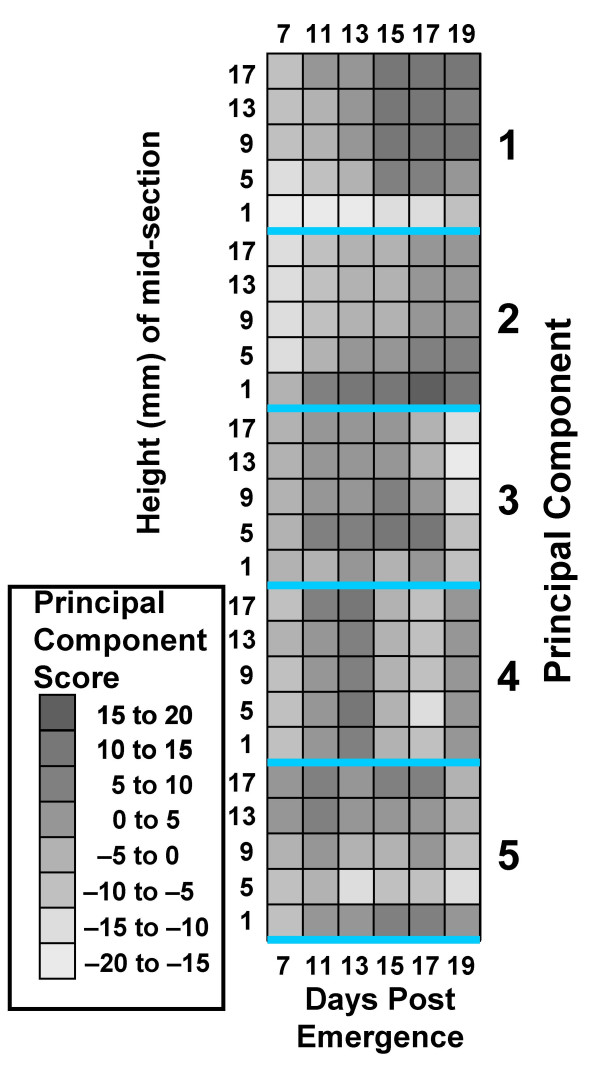
**Principal component scores during a rice plant developmental period bridging first tillering**. The scores (categorized by value using a grey-scale as indicated in the legend) of Principal Components 1 to 5 (Panels 1 to 5, respectively) are plotted against the progression in sampling of days post-emergence (horizontal axis) and the height of the sampled tissue section (as height [mm] of mid-section – the vertical axis of each panel). Rice plants have a basal meristem, so an increase in mid-section height is also a progression in development. The principal components are of a standardized, centered metabolite space from an analysis of a comprehensive metabolomics dataset. Principal Components 1, 3 and 5 show a pattern of change relative to both developmental variables (axes), while Principal Component 4 and Principal Component 2 (if the influence of the 1-mm mid-section height samples are ignored) vary mainly with days post-emergence and are probably influenced by environment more than development. Each value is the mean of three replicates, each of which pooled sections representing 50 different plants.

The first pass at interpreting the principal components involved plotting the scores against the two developmental variables of days post-emergence and height of the sample's mid-section (Figure 1). If a principal component was strongly influenced by development, then its score would be expected to demonstrate a pattern of change with respect to both developmental variables. Such a pattern was observed in the plots of Principal Component 1, 3, and 5 scores (Figure 1). If a principal component was strongly influenced by environment, independently of development, then the scores would be expected to demonstrate a pattern of change relative to days post-emergence but not height of the tissue section at sampling. An example of a possible environmental variation that could have an influence only on days post-emergence would be change in photosynthetic radiation intensity on the day of harvest due to variation in cloud cover. Such a pattern was observed in the plots of Principal Component 4 (and probably also Principal Component 2, if the strong influence of the most basal tissue location is temporarily disregarded) (Figure 1). The tendency of the main influence on principal component scores' variations to alternate among two main categories (possibly developmental and environmental) of influences is not unusual (for another example, see Tarpley et al. [15], in which there was demonstrated alternation between physiological and environmental influences).

### Patterns in metabolite loadings on Principal Component 1

Patterns in metabolite loadings on Principal Component 1 suggested some metabolite variation could be interpreted via well-known metabolism. For example, Ireland [16] discusses the primary routes of nitrogen flow in amino acid synthesis in plants. A number of the involved amino acids were also relatively strong – positive or negative – contributors (loaders) on the Principal Component 1 of our study. About half of the relatively strong (top 8 or 9 positive or negative loaders of the 155 metabolites that were identified with a standards library, and remaining after removal of some members of highly correlated sets of metabolites) were amino acids, including those indicated by Ireland, indicating that patterns of metabolites observed in relation to plant developmental or environmental factors can sometimes be related to well-known metabolism. For Principal Component 1 in this study, several of the relatively strong positive loaders (serine, glycine, alanine, aspartate) are fairly close in metabolic space to glutamate, which is a relatively strong negative loader, and thus somewhat distant from them in our metabolite space. Glutamate and aspartate, for example, are separated by a single metabolic step – the glutamate:oxaloacetate aminotransferase [16].

These same four metabolites opposing glutamate in the Principal Component 1 loading in metabolite space (alanine, aspartate, and the glycine/serine ratio) have been proposed as a biomarker metabolite set of the relative rate of photorespiration in many C_3 _crop species based on physiological understanding [17,9]. Photorespiratory activity usually increases with advancement in leaf development [18,19], and would be expected to increase during the developmental period in this study. The high positive loadings of the photorespiration markers on Principal Component 1 supports this expectation because Principal Component 1 scores tend to increase with development, thus these high positive loaders are increasing in relative concentration during development also. The metabolic links among these amino acids, or of the biomarker metabolite set capturing their behaviour along with those of other metabolites in more dimensions of the metabolite space, will be of interest in interpreting the metabolite variance present in tiller initiation and early development in rice.

### Metabolite clustering based on ranked principal component loadings

Metabolite selection was initiated via K-means clustering into 27 clusters based on ranked loadings on the three top principal components. Clusters representing 20 of these 27 combinations were found. From 17 of these clusters, representative metabolites were selected based foremost on their proximity to the center of the cluster and nextmost on their perceived commonality as a metabolite. For four of the remaining ten desired combinations, a metabolite from a neighboring cluster was considered sufficiently close to be useful when it had loadings on the first two principal components categorically identical to that sought and with "near-miss" location in loadings on Principal Component 3. No representative metabolites were found for the remaining six combinations. The strongest pattern in common among the six unfilled combinations is that only one has a strong negative loading on Principal Component 1. In other words, because of the tendency of Principal Component 1 scores to increase with development (grasses have basal meristematic tissue, so an increase in mid-section height is also a progression in development) (Figure 1), a representative metabolite could almost always be found in this study when a requirement for that metabolite was to possess a large specific proportional decline in its concentration during development relative to other metabolites. The resultant set of metabolites and the combinations they represent are illustrated in Table 1. An additional data file (see Additional file: 1) lists the metabolites that were used in the principal component analysis and subsequent clustering, and have been at least partially or tentatively identified. The actual ranks based on loading values on the top three principal components are provided.

The distribution of the Pearson correlation values of pairwise comparisons among the selected biomarker metabolites was determined. The systematic method used to select the biomarker metabolites would be expected to yield a range of Pearson values. For example, a metabolite picked from a cluster of metabolites with strong negative loadings on Principal Components 1, 2, and 3 (i.e., trehalose) would be expected to have a fairly positive correlation with citrate (negative on Principal Components 1 and 2, little loading on 3), not much correlation with galactose (not much loading on Principal Components 1 and 2, negative on 3), and a strong negative correlation with uracil (positive loader on all three Principal Components). The mean Pearson value was 0.06 ± 0.06 (95% confidence interval) and the dispersion was 0.41 ± 0.04 (95% confidence interval) (for comparison, a normal distribution would have a mean of 0 and a dispersion of 1, but remember that a non-pathological distribution of correlation values cannot have a dispersion approaching 1 because the correlation values are always between -1 and 1), indicating a satisfactory – relatively independent – distribution of biomarker metabolites based on their correlations.

### Interpretation of physiological relevance of biomarker metabolites in relation to their principal component loadings

The biomarker metabolites presented here were selected partially based on their range of loadings on the top three principal components, and any interpretation of their physiological relevance starts with an examination of physiologically relevant groups of them contributing in common to a particular principal component.

The Principal Component 1 score tends to increase with development (Fig. 1), thus the relatively high proportion of amino acids among the biomarkers with strong negative loading on Principal Component 1 (*gamma*-amino butyric acid, glutamate, leucine, lysine and phenylalanine) is consistent with other observations of a decrease in amino acid contents of leaves during development [20].

The biomarkers present in the tricarboxylic acid cycle (malate, succinate and citrate) are all strong negative loaders on Principal Component 2. This component tends to vary more with days post-emergence than with the more strictly developmental variable of height of sampling of the tissue. These results suggest the tricarboxylic acid cycle is subject to coarse control in response to the environmental factor/s influencing Principal Component 2 values.

A strong opposition exists between the positive- and negative-loading biomarkers on Principal Component 3 with respect to the nitrogen content of the metabolites. Nearly all of the positive-loading biomarker metabolites are nitrogen-containing – glutamate, leucine, phenylalanine, pyroglutamate, thymine, uracil and valine; the sole exception is *trans*-aconitate. *Trans*-aconititate is the stable form of aconitate and is capable of being formed from *cis*-aconitate [21]. Nearly all of the negative-loading biomarker metabolites are sugar or organic acid, non-nitrogen compounds – galactose, mannose, trehalose, carbonate, malate, oxalate and shikimate; the sole exception is *gamma*-amino butyric acid (GABA). Among the higher plants, GABA is the most widely distributed of the amino acids in which the amino group is not in the alpha position [22].

### Reliability of the biomarker set to represent the pattern of metabolite variation observed among the tissues

If the resultant set of biomarker metabolites captures much of the metabolite variance in development as obtained through the metabolomic profiling, then we should be able to "flip" the analysis around and detect natural patterns of tissue relationships that relate to development, and possibly environment, based on their biomarker metabolite concentrations.

**Figure 2:**
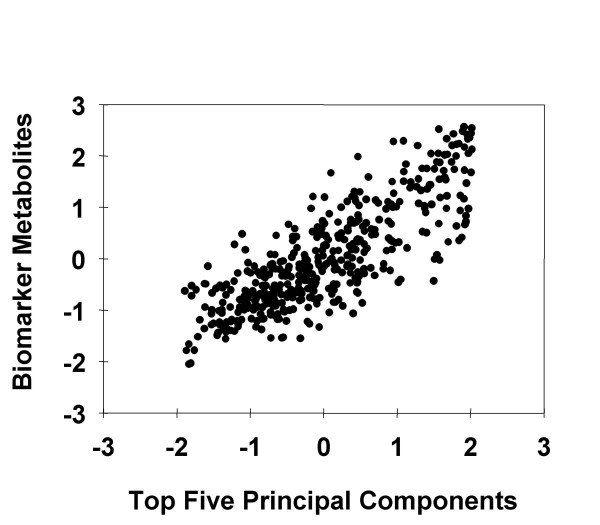
**Plot of correlations among samples based on biomarker metabolites vs. based on principal component scores**. The biomarker metabolite set does a reasonable job of mimicking the pattern among the sampled tissues based on the top five principal components. The pattern among the tissues with respect to their metabolite composition is discerned by the set of all pairwise Pearson correlation values among the tissues. This "correlation measure" of the pattern among the tissues was applied using two different sets of markers. The set represented by the horizontal axis used the scores of the top five principal components from the analysis of the comprehensive metabolomics dataset. The set represented by the vertical axis used the biomarker metabolite concentrations. These metabolite concentrations are standardized and centered because our study was mainly interested in the magnitude and pattern of the variation in the metabolites during development. The correlation values plotted in the figure have been transformed to a *Z*-scale to bring out the accuracy (slope of a fitted line would be near 1 with an intercept near 0) in the ability of the biomarker metabolite set to mimic the pattern among the tissues, and with reasonable precision (r = 0.82).

In order to evaluate the biomarker metabolite set for ability to consistently detect patterns in metabolite distributions among the tissues during development, we compared the set of all correlations among the sampled tissues based on the standardized and centered concentrations of the 21 biomarker metabolites vs. the set of all correlations based on the values of the five principal components that explained most (83%) variance in the original metabolomics data set. Figure 2 is a scatter plot of the pairs of values for these two data sets. These metabolite concentrations are standardized and centered in the figure because our study was mainly interested in the magnitude and pattern of the variation in the metabolites during development. The correlation values plotted in the figure have also been transformed to a *Z*-scale to bring out the accuracy (slope of a fitted line would be near 1 with an intercept near 0) in the ability of the biomarker metabolite set to mimic the pattern among the tissues, and with reasonable precision (r = 0.82).

The biomarker metabolite set was determined to be reliable (*P *= 0.001) in capturing the metabolite-based relationships among tissues present in this particular study of the advent of tillering in rice. We chose to not try to explain any more of the variance of the original dataset because our objective was to develop a biomarker metabolite set to detect patterns among the tissues during development, but we could not, using biological reasoning, explain any of the principal components after Principal Component 5 in terms of patterns among tissues during development. Thus, using only the first five principal components served as a way of filtering out noise [23] in the comprehensive dataset. This helps avoid overfitting of the data [24].

### External validation of the biomarker metabolite set

The proposed biomarker metabolite set gains value if used in comparative screening, but its use in comparative situations requires some confidence in its transferability to different situations. Partial confidence is provided by the internal validation described in the above section, which indicates the set was well-constructed, and also by some natural groupings of metabolites associated with the detected principal components. Full confidence, however, requires a demonstration that the set is transferable. An external validation was developed by re-analysing the data of Sato et al. (2004) [25], which is a capillary electrophoresis (CE) – mass spectrometer: CE-diode array detector metabolomics study of rice leaves, with an emphasis on the day/night transition (their Table 2). Pairs of metabolites were identified, in which one member of a pair is a biomarker metabolite that they also measured in their study, and the other member of a pair was a metabolite measured by Sato et al. for which a metabolite had a definitive pairing with the biomarker metabolite. The changes in the day/night ratio in metabolite concentration were compared for the members of each pair that could be constructed. For the six pairs that could be used in the validation, 4 predicted a higher day/night ratio with a higher day/night ratio being observed for each of them, 1 predicted a lower day/night ratio with a lower ratio being observed for it, and one predicted no change in day/night ratio with a higher ratio being observed. The results indicate that the biomarker metabolite set is likely (*P *= 0.05) to be useful in some other situations, for which these primary and central metabolites would be expected to change in a highly regulated manner, because the tested biomarkers worked well as predictors in the different environmental situation of the day/night transition in leaves.

### Example output from comparative screening of biomarker metabolite patterns

**Figure 3:**
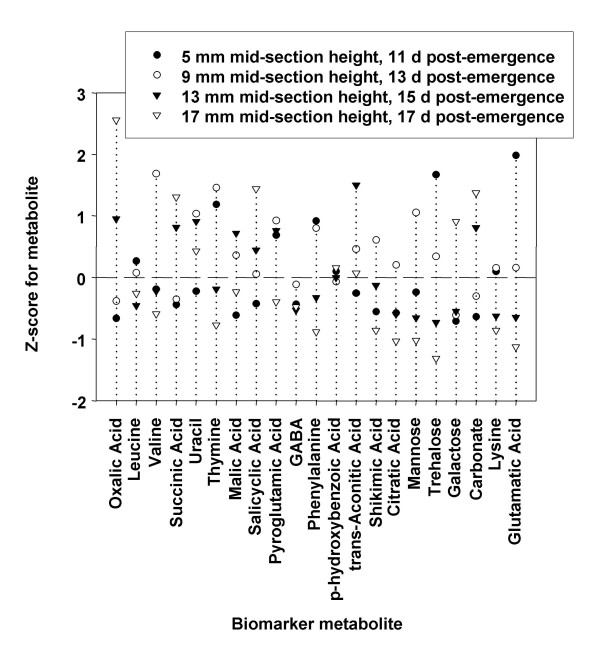
**Magnitude and pattern of variation in biomarker metabolite concentrations in samples ranging in development**. Four of the tissue samples representing a developmental range are profiled with respect to biomarker metabolite variation. The samples progress in height at mid-section of the sampled tissue and in days post-emergence, thus they represent a cross-section of the larger set of tissue samples. The biomarker metabolites are listed along the horizontal axis, and each dot plot shows the *Z*-scores for the biomarker metabolite concentration. The metabolites vary a lot in absolute concentration, so the *Z*-score is used to equalize the overall variation in concentration of the metabolites during the study. Thus the figure shows the pattern and magnitude of the variation among the presented tissues, but also the amount of this variation relative to that of the metabolite concentration for the whole study. For example, oxalic acid and glutamate both have a fairly wide range of *Z*-scores for these samples, although the pattern of variation is opposite.

A possible way of presenting the results of a biomarker metabolite study is provided in Figure 3, in which four of the tissue sections representing a developmental range in the tillering study are profiled with respect to biomarker metabolite variation. Clearly, these biomarkers of metabolite change in the tillering study change in definable ways. Some (such as several of the organic acids) tend to increase in concentration during development, others (leucine, phenylalanine, trehalose, glutamate) decrease, and others exhibit a more complex pattern. The combination of these patterns reliably reflects the changes in the comprehensive metabolomic profile, and provides a fresh view of the biology of this developmental event. The presentation of metabolite variation based on biomarker variation allows viewing in a single chart whereas the variation of the original 332 metabolites (actually those remaining after the removal of some members of highly intercorrelated sets of metabolites) cannot be easily captured in a single chart. In contrast, the presentation of the variation in only one or a few metabolites fails to capture the broader patterns of change in composition that the presentation of the variation in the biomarker metabolites provides. The distortions in the shape (the relationships of the biomarker metabolites to each other based on their patterns of variation) in the biomarker metabolite space can be captured graphically and/or mathematically and evaluated as an approximation of the change in the comprehensive set of metabolites.

### Applicability of biomarker metabolite set for comparative screening

Tillering is an important, well-regulated developmental event in cereal crops and many other grasses. The ability to capture the metabolite variance of this developmental profile using a small set of common metabolites as biomarkers suggests the ability to capture a large portion of it in other plant developmental events using the same set of biomarker metabolites. These metabolites are typically involved in heavily regulated metabolic activities, and it comes as no surprise that they can be useful as biomarkers in plant development. Differences in the use of various metabolic pathways need to occur in different developmental or growth response events, but these are differences in degree. Some of these differences can help modulate the different developmental or growth response events [9]. The interrelationships among the biomarker metabolites would be expected to change in consistent ways in response to change in development, genotype and environment. Comparisons of the changes in the interrelationships of the biomarkers (of the stretches and tucks – the distortions – in the biomarker space) under various conditions will provide new information about plant physiological response in development and in response to environment. The biomarker metabolite set cannot be optimal for any set of conditions, but is likely to be fairly robust in capturing physiologically real differences among them, while being responsive to eventual metabolic interpretation.

## Conclusion

Variation in crop development due to genotype and environment greatly impacts yield, yet the community's understanding of the quantitative biochemical variation in plant development is small.

This paper has presented an approach to developing a biomarker metabolite set that captures much of the metabolite variation present in a comprehensive metabolomics set of a plant developmental event, tillering, in rice. The resulting biomarker set is intended to provide some of the advantages of a metabolomics approach and of the use of one or a few diagnostic metabolites.

The approach uses simple and commonly available multivariate statistics, namely principal component analysis and K-means clustering, to assist with the biomarker metabolite selection. The resulting set of 21 biomarkers was shown to be reliable in capturing the metabolite variance in the comprehensive metabolomics study and valid for predicting metabolite changes observed in another metabolomics study, while capturing variation that has reasonable potential to be related to well-known metabolism.

The selection of the biomarker metabolites was further constrained to primary or central or common metabolites. Because the selected metabolites are common among diverse organisms and often have specific assay procedures already developed (e.g., Bergmeyer [26], Passoneau and Lowry [27], Gibon et al. [4], and Kiianitsa et al. [28]), the biomarker set is amenable to future assay via high-throughput technologies, and in application to diverse situations. The biomarker metabolite set can serve as a basis for comparative screening of metabolite patterns of plant developmental periods, of plant response to specific environmental factors, or of genotypes in set conditions.

## Methods

### Culture of plants and initial preservation of tissue samples

Seedlings of rice cv. IR-36 were grown in a black clay soil, typical of the area's rice fields, in flats in the greenhouse under typical temperature and supplemental lighting regimes at the Texas A&M Agricultural Research and Extension Center at Beaumont, Texas, USA. Nitrogen fertilizer was applied as urea at the 2–3 leaf stage. Sampling started one week after emergence and continued at two- to four-day intervals for a total of five dates over a 10-day period. Separate sets of seedlings were used at each sampling date, thus avoiding injury to existing plants. The seedlings developed to about the 3-leaf to 5-leaf stage during this interval. All samples were collected, processed and stored between 1000 and 1400 h CDT (near solar mid-day). The soil was washed off the seedlings within five minutes, and the seedlings placed with roots in tap water until dissection. This is an established procedure for maintaining rice plant integrity for short periods during sampling.

Seedlings were sectioned in 2-mm intervals along the developing culm, starting at the base of the plant and continuing for a total of ten sections. At this developmental stage, 2-mm sections are thick enough to ensure that the large majority of cells are not disrupted from the slicing action of the new ethanol-cleansed razor blades. This minimizes injury response. Each replicate contained tissue from an average of 50 seedlings, i.e. 2-mm sections from the same culm position for each of 50 seedlings. Slow-growing seedlings were avoided. The obtained sections were plunged within a few seconds into liquid nitrogen until all sections were collected. Sections were then stored at -80°C in nitrogen-purged vials until lyophilized for use in the metabolomics procedures. There were three replicates, each with sections from 50 seedlings.

Only sections of positions 1, 3, 5, 7 and 9 from the base of the plant were used for the metabolomics. The sections were collected along two gradients: along the culm and during development.

After lyophilization (Labconco Freezone 6, Kansas City, Missouri, USA), the samples were capped in amber glass vials under nitrogen and then sealed externally around the cap rim with polyethylene homopolymer film (Parafilm M; Pechiney Plastic Packaging, Neenah, Wisconsin, USA) to further minimize gas penetration prior to shipment to Noble Foundation laboratories for metabolomic analysis.

### Extraction, derivatization, and instrumental analysis

A total of 6.02 mg (± 0.02 mg) of the lyophilized pulverized tissue was weighed into 3.7-mL (1 dram) vials containing teflon inlays. Metabolite extractions were performed by adding 1.5 mL chloroform containing 5 mg/L phenanthrene internal standard and 1.5 mL bottled water containing 25 mg/L ribitol internal standard, followed by vortexing for 1 min. The samples were incubated at 50°C for 1 h with shaking and placed at -20°C overnight. The samples were again incubated at 50°C for 4 h with shaking. The samples were then sonicated 30 s and centrifuged in a swinging bucket rotor at 2,900 × *g *for 30 min. Aliquots of 1.2 mL were taken from both the polar and lipophilic layers and transferred to 2.0 mL autosampler vials (Agilent, Palo Alto, California, USA) with teflon/silicon septa. The polar layer was dried in a speed vac (Savant, Albertville, Minnesota, USA) for 4 h, and the lipophilic layer dried under a stream of compressed nitrogen for 2 h. Extracts were stored at -20°C until ready for analysis. Both the polar and lipophilic extracts were analyzed.

Dried polar extracts were prepared by methoximation in 120 μl of 15 g/L methoxyamine hydrochloride in pyridine at 50°C for 4 h followed by a brief sonication (<30 s) to dislodge any pellet. Samples were derivatized by adding 120 μl N-methyl-N-(trimethylsilyl)trifluoroacetamide) +1% trimethylchlorosilane followed by incubation at 50°C for 1 h.

Dried lipophilic extracts were prepared by transmethylation of fatty acid and lipids. Dried extracts were dissolved in 100 μl chloroform and 300 μl methanol containing 1.25 M HCl and then incubated at 50°C for 24 h. Samples were then dried under a stream of nitrogen for 2 h. Dried transmethylated samples were resuspended in 70 μl pyridine and derivatized in 30 μl N-methyl-N-(trimethylsilyl)trifluoroacetamide) +1% trimethylchlorosilane at 50°C for 1 h.

Derivatized metabolite mixtures were analyzed using a Hewlett Packard 6890 gas chromatograph, 5973 mass selective detector, and 6890 series injector. The integrated system was operated under HP Chemstation (Agilent). Polar samples were analyzed by injecting 1 μl with a split injection ratio of 5:1; lipophilic samples of 1 μl were analyzed using a 1:1 split injection ratio. All samples were injected in duplicate. Analyses were performed using a 60-meter DB-5MS capillary separation column (J&W Scientific, Palo Alto, California, USA). Injection temperature was 280°C, interface 280°C. Separations were achieved using the following temperature program: 3 min isothermal heating at 80°C, followed by a 5°C min^-1 ^oven ramp to 315°C, and a final isothermal heating at 315°C for 14 min for polar and 12 min for lipophilic samples. Mass spectra were recorded at 2.48 scans s^-1 ^with a mass scanning range of 50 to 650 m/z. Each run required approximately 70 min including machine equilibration time.

Metabolite identifications were determined using GC-MS spectral database matching against the current National Institute of Standards and Technology library (NIST02) and a Noble Foundation in-house custom database focused on plant metabolites. Standards for construction of the in-house library were prepared by methoximation and derivatization as described above. The Automated Mass Spectral Deconvolution and Identification Software (AMDIS) (National Institute of Standards and Technology (NIST), Gaithersburg, MD) was utilized for library construction and metabolite identification in the raw metabolite profiles.

The GC-MS chromatographic data alignment was performed according to Duran et al. [29]. Selected ions were extracted and aligned from raw metabolomic data files using a custom Perl script. This provides a more comprehensive interrogation of the data and is capable of resolving coeluting chromatographic peaks based on the underlying mass data.

### Correlation among metabolites

The correlations between pairs of metabolites were evaluated as Pearson correlation coefficients [30]. One or more metabolites from highly intercorrelated sets of metabolites were omitted from the dataset when necessary to ensure subsequent calculations did not involve singular matrices [31]. Singular matrices do not provide unique solutions with many multivariate statistical methods.

### Principal component analysis

Principal components in standardized centered metabolite space were determined. This analysis included metabolites that were not matched against a standards library, but did not include those omitted to avoid singular matrices. The analysis was performed in MathCad 2001 (MathSoft, Inc., Cambridge, Massachusetts, USA) using the matrix manipulations described by Pielou [23]. Although there are methods available that can assist in determining which principal components to retain (e.g., scree analysis), the cut-off was made based on the observations that other principal components neither explained much of the variance nor exhibited any pattern in metabolite loadings that could be easily related to known metabolism, or as a response to developmental or environmental variables [32].

### Metabolite selection via K-means clustering

Individual metabolites were selected to provide a spread of variation in loadings on the first three principal components, while being potentially easy to assay. Metabolite selection involved K-means clustering of the ranked loadings on the three top principal components, followed by individual selection of promising metabolites from the clusters. The K-means clustering was performed using Cleaver (Classification of Expression, Array Version 1.0) software available through the site for microarray analysis maintained by the Helix Bioinformatics Group at the Stanford School of Medicine [33]. The analysis sought 27 clusters using the Euclidean distance metric. This set-up maximized the potential to obtain clusters representing all possible combinations of strong positive loading, weak loading, and strong negative loading elements for each of the three principal components. In other words, 3 loading strengths × 3 principal components, taken three at a time = 27 combinations for which a representative metabolite was desired. Representative metabolites were selected based foremost on their proximity to the center of the cluster and then on their perceived commonness as a metabolite. For the remaining clusters, a metabolite from a neighboring cluster was considered sufficiently close to be useful when it had loadings on the first two principal components categorically identical to that sought and with "near-miss" location in loadings on Principal Component 3. If no sufficiently representative metabolite could be found with this approach, then no further effort was made and such clusters remained unrepresented.

### Reliability of the biomarker set to represent the pattern of metabolite variation observed among the tissues

In order to evaluate the biomarker metabolite set for ability to consistently detect patterns in metabolite distributions among the tissues during development, the set of all pair-wise Pearson correlations [30] among the sampled tissues based on the standardized and centered concentrations of the biomarker metabolites were obtained. The equivalent set of all pair-wise correlations was obtained based on the unranked values of the five principal components that explained most (83%) variance in the original metabolomics data set. The reliability of the biomarker metabolite set to capture the metabolite variation present among the tissues in development was obtained by comparing the two sets of correlation values using the methods of Fisher [30]. There are 435 pairs of values to compare, but these are not truly independent variables, so the reliability was analyzed with 28 degrees of freedom (30 tissue samples minus 2 degrees of freedom) to account for the lack of independence among pairs of correlation values.

### External validation of the biomarker metabolite set

An external validation analysis was developed by re-analysing the data of Sato et al. (2004) [25], which is a capillary electrophoresis (CE)- mass spectrometer: CE-diode array detector metabolomics study of rice leaves, with an emphasis on the day/night transition (their Table 2). Pairs of metabolites were identified, in which one member of a pair was a biomarker metabolite that they also measured in their study, and the other member of a pair was a metabolite measured by Sato et al. for which a metabolite had a definitive pairing with the biomarker metabolite. The changes in the day/night ratio in metabolite concentration were compared for the members of each pair that could be constructed, and the directions of changes in the biomarker metabolites were used to predict the changes in the other metabolites. The average percentage change in concentration of the metabolites used in this validation test was considered fairly small at 51% of the night-time value, thus justifying the use of the measure of the ability of the biomarkers to indicate the direction of change in concentration of the respective predicted metabolites in the day/night transition as a reasonably powerful validation test of the biomarkers' ability to mark broad changes in the other metabolites.

## Authors' contributions

Culture of plants, sampling of tissues and initial preservation of tissue samples (THK, LT); Extraction, derivatization, and instrumental analysis through and including mass spectral interpretation and metabolite identification (ALD; LWS); Data analysis related to development of the biomarker metabolite set; planning and writing the manuscript (LT). All authors read and approved the final manuscript.

## Supplementary Material

Additional File 1 **Ranked principal component loadings of identified metabolites**. List of metabolites that were used in the principal component analysis and subsequent clustering, and have been at least partially or tentatively identified. The actual ranks based on loading values on the top three principal components are presented. The selected biomarker listings are bolded. When a biomarker has multiple metabolite entries, then these entries are matched through use of a non-black font color. Click here for file

## Figures and Tables

**Table 1:** The selected biomarker metabolites, and the combinations of loadings on principal components that they represent. The set of biomarker metabolites selected to capture much of the variance in metabolite composition of a rice tillering event is listed. The selection procedure constrained the biomarker metabolites to represent variation in loading on the top three principal components in the standardized centered metabolite space of an analysis of a comprehensive metabolomics dataset. The loading of the individual metabolite on the principal components is symbolically represented: high positive loading (**POS**), weak loading (**---**), or high negative loading (**NEG**).

**Biomarker metabolite**	**Principal Component 1 loading**	**Principal Component 2 loading**	**Principal Component 3 loading**
Trehalose	**NEG**	**NEG**	**NEG**
Citric Acid	**NEG**	**NEG**	**---**
Glutamic Acid	**NEG**	**NEG**	**POS**
Mannose	**NEG**	**---**	**NEG**
Phenylalanine	**NEG**	**---**	**POS**
gamma-Aminobutyric Acid (GABA)	**NEG**	**POS**	**NEG**
Lysine	**NEG**	**POS**	**---**
Leucine	**NEG**	**POS**	**POS**
Shikimic Acid	**---**	**NEG**	**NEG**
Succinic Acid	**---**	**NEG**	**---**
Pyroglutamic Acid	**---**	**NEG**	**POS**
Galactose	**---**	**---**	**NEG**
Valine	**---**	**---**	**POS**
p-Hydroxybenzoic Acid	**---**	**POS**	**---**
Thymine	**---**	**POS**	**POS**
Malic Acid	**POS**	**NEG**	**NEG**
Salicylic Acid	**POS**	**NEG**	**---**
Oxalic Acid	**POS**	**---**	**NEG**
trans-Aconitic Acid	**POS**	**---**	**POS**
Carbonate	**POS**	**POS**	**NEG**
Uracil	**POS**	**POS**	**POS**
